# Long Time CO_2_ Storage Under Ambient Conditions in Isolated Voids of a Porous Coordination Network Facilitated by the “Magic Door” Mechanism

**DOI:** 10.1002/advs.202307417

**Published:** 2023-11-20

**Authors:** Terumasa Shimada, Pavel M. Usov, Yuki Wada, Hiroyoshi Ohtsu, Taku Watanabe, Kiyohiro Adachi, Daisuke Hashizume, Takaya Matsumoto, Masaki Kawano

**Affiliations:** ^1^ Department of Chemistry School of Science Tokyo Institute of Technology 2‐12‐1 Ookayama Meguro‐ku Tokyo 152–8550 Japan; ^2^ Central Technical Research Laboratory ENEOS Corporation 8 Chidoricho Naka‐ku, Yokohama Kanagawa 231–0815 Japan; ^3^ RIKEN Center for Emergent Matter Science (CEMS) 2‐1 Hirosawa Wako Saitama 351‐0198 Japan

**Keywords:** carbon‐neutral society, gas adsorption, machine learning potential, metal–organic frameworks, X‐ray structure analysis

## Abstract

A coordination network containing isolated pores without interconnecting channels is prepared from a tetrahedral ligand and copper(I) iodide. Despite the lack of accessibility, CO_2_ is selectively adsorbed into these pores at 298 K and then retained for more than one week while exposed to the atmosphere. The CO_2_ adsorption energy and diffusion mechanism throughout the network are simulated using Matlantis, which helps to rationalize the experimental results. CO_2_ enters the isolated voids through transient channels, termed “magic doors”, which can momentarily appear within the structure. Once inside the voids, CO_2_ remains locked in limiting its escape. This mechanism is facilitated by the flexibility of organic ligands and the pivot motion of cluster units. In situ powder X‐ray diffraction revealed that the crystal structure change is negligible before and after CO_2_ capture, unlike gate‐opening coordination networks. The uncovered CO_2_ sorption and retention ability paves the way for the design of sorbents based on isolated voids.

## Graphical abstract

A coordination network selectively captures and traps CO_2_ despite lacking pore accessibility. Neural Network Potential (NNP) simulations show that CO_2_ diffusion is enabled by formation of transient channels through slight structural adjustments, which are dubbed “magic doors”. The presence of a guest‐dependent activation energy barrier necessary for this process can explain the adsorption selectivity and CO_2_ retention ability of this network.

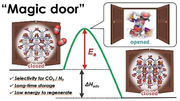

## 1 Introduction

To achieve a carbon‐neutral society, there is an urgent need to develop materials for CO_2_ capture and storage. Porous coordination networks (PCNs), also known as metal–organic frameworks (MOFs), which are composed of organic multidentate ligands combined with metal ions, have attracted attention as promising candidates because of their readily modifiable structures and tunable pore environments. Previous efforts to improve the affinity of networks for CO_2_ focused on increasing the number of point interactions with the adsorbed molecules by incorporating open metal sites,^[^ ^1^, ^2^ ^]^ amino groups,^[^ ^3^, ^4^, ^5^ ^]^ or inorganic fluorinated anions^[^ ^6^, ^7^ ^]^ into their structures. Additionally, PCNs with flexible structures were also explored for this application. They rely on structural transformations that are induced by pressure changes and can be selectively triggered by CO_2_, including gate opening^[^ ^8^, ^9^, ^10^, ^11^, ^12^, ^13^, ^14^ ^]^ and rotation dynamics of ligands.^[^ ^15^, ^16^, ^17^ ^]^

Although there has been considerable progress in improving CO_2_ uptake capacities and selectivity,^[^ ^18^, ^19^, ^20^, ^21^, ^22^ ^]^ the possibility of its long‐term storage inside PCNs under ambient conditions has received comparatively less attention since the interactions between the adsorbed gas and the network are typically facilitated by weak intermolecular interactions (physisorption). Therefore, to prevent gas diffusion outside, functional groups that can form stronger covalent bonds with CO_2_ molecules (chemisorption) need to be introduced. However, because of the high stability of the resultant chemical bonds, CO_2_ desorption and sorbent regeneration can be energy intensive, as in the case of amino‐based liquids which are commonly used to capture CO_2_ in industrial settings.

In this study, we present an alternative approach for trapping CO_2_ inside PCNs. An interpenetrated network containing isolated voids between two adjacent nets was constructed. Even though these voids cannot be directly accessed, stretchable transient channels resembling “magic doors” could appear, enabling the material to adsorb CO_2_ and exclude N_2_. Once confined inside, CO_2_ did not exhibit any strong interactions with the pore interior and the same mechanism was impeding its diffusion to the outside. As a result, the network could retain CO_2_ for more than one week while being kept on a bench.

## 2 Results and Discussions

**Figure 1:**
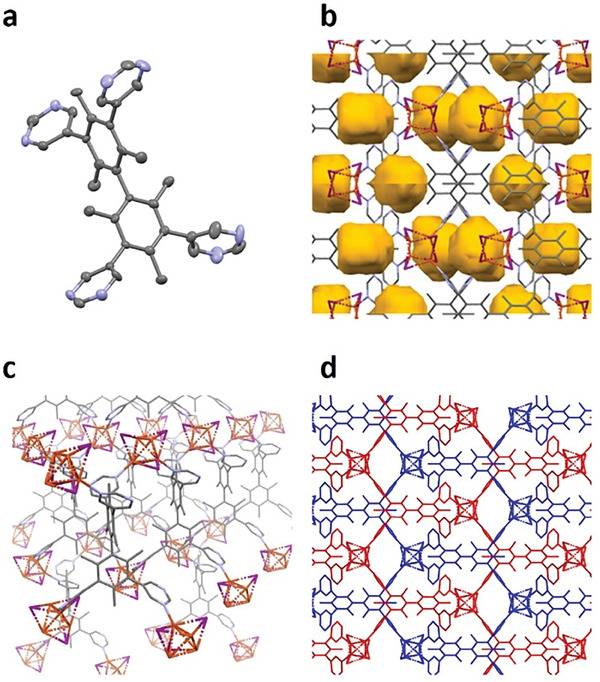
a) Single crystal structure of **L**. The structure of network **2**, b) the voids outlined in dark yellow, c) a single diamondoid‐type net, and d) two interpenetrated nets represented by different colors. C – grey, N – blue, Cu – orange, and I – purple, hydrogen atoms were omitted for clarity.

To obtain the desired material, a ligand (**L**) based on a pseudo‐tetrahedral bimesityl skeleton^[^ ^23^, ^24^ ^]^ decorated with four pyrimidine groups was developed (**Figure** **1**a; Figures S2 and S3, Supporting Information). Because of its steric bulk and orientation of nitrogen atoms around pyrimidine rings, the coordination directions are bent at an angle against the center–vertex axes. Therefore, the coordination of this ligand was expected to create PCNs with highly compact convergent structures featuring small pores. The reaction of **L** with CuI in the presence of KI and PPh_3_ in the CH_3_CN/H_2_O/EtOH solvent mixture under solvothermal conditions yielded two distinct crystal morphologies, yellow plates and yellow prisms. Single crystal X‐ray diffraction analysis (SCXRD) revealed that the former crystals corresponded to a 2D network with the molecular formula of {[(Cu_2_I_2_)(**L**)]·solvent}_n_ (**1**). On the other hand, the prisms were a different polymorph, {[(Cu_4_I_4_)(**L**)]·solvent}_n_ (**2**), with a 3D interpenetrated structure. The two PCNs always crystallized together as a mixture during the synthesis. However, because of differences in their crystal densities, 1.782 and 2.086 g cm^−3^ for **1** and **2**, respectively, pure samples could be obtained by the density separation method.^[^ ^25^ ^]^

The structure of network **1** consisted of Cu_2_I_2_ dimers connected by four pyrimidine groups from four separate ligands forming staggered 2D layers (Figure S4, Supporting Information). In comparison, network **2** had a diamondoid‐type structure containing Cu_4_I_4_ cubane clusters as connector nodes (Figure 1b−d). Because each Cu ion coordinated to only one nitrogen atom in each pyrimidine ring, the resultant network was highly distorted limiting the degree of interpenetration to only two nets. This material possessed closed pores with dimensions of 5.2 × 4.9 × 4.9 Å. However, the size of the maximum aperture between each pore was only 2.4 × 1.5 Å, which was too small for any molecule to pass through. Nevertheless, acetonitrile molecules were present in the pores of the as‐synthesized crystal of **2** (Figure S5, Supporting Information), which could be removed by heating at 473 K for 12 h under vacuum without loss of crystallinity, suggesting that some diffusion pathways could exist for the guest escape to occur (vide infra). The crystallinity of network samples was assessed by powder X‐ray diffraction (PXRD). The measured patterns (Figures S8 and S9, Supporting Information) matched closely with the simulated patterns from the single crystal structures, thus confirming their phase purity. Thermogravimetric analysis (TGA) and differential scanning calorimetry (DSC) were performed, revealing that networks **1** and **2** were stable up to 400 °C at which point the ligand began to decompose (Figure S10a–c, Supporting Information). A smaller weight decrease was observed at ≈250 °C, which was due to the evaporation of solvent (MeCN) from the pores.

**Figure 2:**
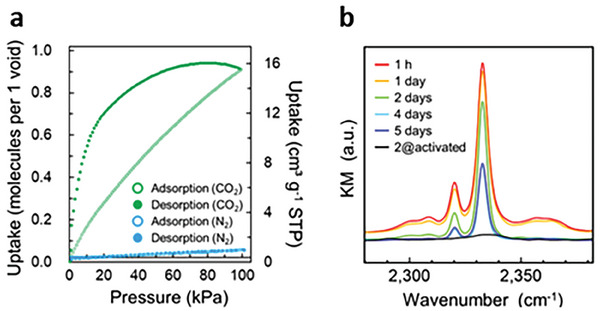
a) Adsorption and desorption isotherms of N_2_ and CO_2_ for **2@activated** measured at 298 K. b) Time‐dependent IR spectra of **2@CO**_**2**_ left in the air.

These results prompted us to investigate the possibility of adsorbing different gases into the two networks. First, N_2_ isotherms measured at 77 and 298 K showed no appreciable uptakes in both structures (**Figure** **2**a; Figures S11 and S12b, Supporting Information). Additionally, network **1** displayed only a negligible uptake capacity for CO_2_ at 298 K (Figure S11a, Supporting Information). These results indicated that the pores in its structure are not accessible to small gas molecules in the measured pressure range. In contrast, network **2** adsorbed CO_2_ at several temperatures, reaching close to one molecule per void, indicating a complete saturation (Figure 2a; Figure S12, Supporting Information). The material could be fully regenerated and reused for up to five cycles without any loss of uptake capacity (Figure S13, Supporting Information). Furthermore, the CO_2_ sorption isotherm showed hysteresis between adsorption and desorption branches, suggesting some degree of guest trapping.^[^ ^8^ ^]^ The Clausius–Clapeyron equation, which is typically employed for calculation of the isosteric heat of adsorption for gases,^[^ ^26^ ^]^ could not be applied to the sorption data of network **2** because of the crossing over of isotherms measured at different temperatures. Unexpectedly, the CO_2_ isotherm measured at 195 K showed almost no uptake (Figure S12, Supporting Information), suggesting that some energy barrier was blocking its entry (vide infra). Since desorption only began at low pressures, it was speculated that the loaded material might retain CO_2_ even if exposed to ambient air. To test this hypothesis, infrared spectroscopy (IR) was performed. The crystals of **2@activated** were sealed in 1 atm of CO_2_ for 1 day ensuring a complete saturation of the available pores to give **2@CO**_**2**_. Its IR spectrum contained a strong absorption band at 2340 cm^−1^, indicating the presence of physisorbed CO_2_ (Figure S14, Supporting Information).^[^ ^27^, ^28^ ^]^ This interpretation was additionally corroborated by the TGA‐DSC measurement, which revealed a gradual weight loss in the 70–200 °C temperature range matching the weight of ≈1 CO_2_ molecule per 1 formula unit of network **2** (Figure S10d, Supporting Information). The crystals of **2@CO**_**2**_ were left in the air while monitoring changes in the IR peak over time. Remarkably, the IR signal was still present after 1 week (Figure 2b). Following the peak evolution with time (Figures S15 and S16, Supporting Information) showed a quick drop in intensity in the first 5 h, which then stabilized at ≈12% of the initial absorption even after > 60 h. To the best of our knowledge, such long‐term trapping ability has not been reported in other coordination networks where CO_2_ interaction is limited to physisorption. As a comparison, the same experiment was performed on the CO_2_‐loaded HKUST‐1 framework ([Cu_3_(BTC)_2_], BTC = 1,3,5‐benzenetricarboxylic acid), however, no IR signal from the adsorbed CO_2_ was detected (Figure S17, Supporting Information). This means that the gas molecules quickly escaped the pores as soon as the powder was exposed to the air and before it could be transferred into the IR cell, thus further emphasizing the unique trapping ability of network **2**.

**Figure 3:**
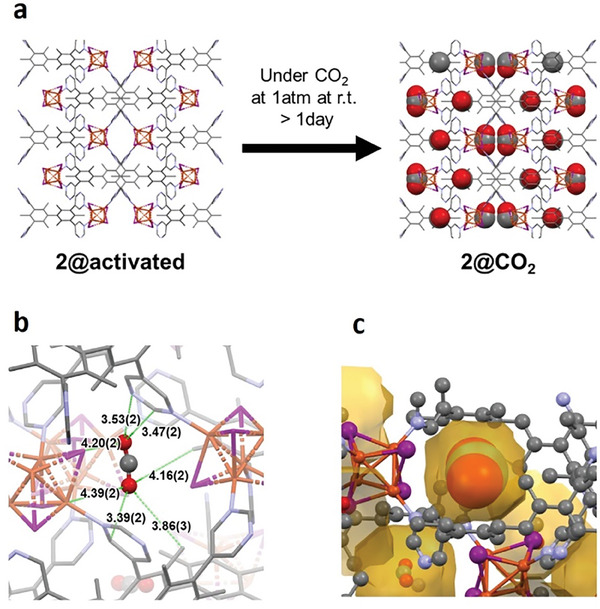
a) Adsorption of CO_2_ into network **2** showing the single crystal structures of **2@activated** and **2@CO**_**2**_. b) Short contacts between network **2** and a CO_2_ molecule. c) CO_2_ orientation inside the pore (dark yellow). C – grey, N – blue, Cu – orange, and I – purple, hydrogen atoms were omitted for clarity.

To elucidate the structural changes accompanying the CO_2_ adsorption and visualize the encapsulated gas molecules, SCXRD analysis was performed. X‐ray diffraction data of **2@activated** and **2@CO**_**2**_ were collected at 90 and 298 K. Comparing the crystal structures before and after CO_2_ adsorption, the network backbone did not experience any changes, regardless of the measurement temperature (**Figure** **3**a). At the same time, the pores remained isolated and inaccessible. The 90 K structure of **2@CO**_**2**_ contained electron density in the center of the void, which was assigned to the CO_2_ molecule (Figure 3b,c). The representative short distances between the network and each atom of CO_2_ were Cu(network)···O(CO_2_) = 4.39(2) Å, I(network)···O(CO_2_) = 4.20(2) Å, C(network) ···O(CO_2_) = 3.39(2) Å, and N(network)···O(CO_2_) = 3.53(2) Å. None of these contacts could be attributed to any bond formation. Therefore, the CO_2_ adsorption into network **2** was facilitated primarily by the weak secondary interactions. These findings were further corroborated by in situ PXRD of network **2** collected in a CO_2_ atmosphere (Figure S18, Supporting Information). The diffraction patterns displayed almost no change after the adsorption, which matched the single crystal results. Interestingly, the opening of channels, as CO_2_ was diffusing through the network (vide infra), also could not be observed in the collected data, indicating that they appeared at random locations in the crystal without the creation of any long‐range order.

The fact that no obvious structural changes were observed in network **2** before and after CO_2_ encapsulation indicated that the aperture between the pores opened only momentarily allowing the guest molecule to pass through. This effect could also be responsible for the lack of N_2_ uptake. To further explore these structural dynamics, the adsorption behavior of **2** was simulated with PreFerred Potential (PFP)^[^ ^29^ ^]^ version 3.0.0 on Matlantis.^[^ ^30^ ^]^ First, the enthalpies of adsorption (Δ*H*_ads_) were calculated. Optimizing structures with and without guest molecules, and comparing their energy states, generated Δ*H*_ads_ values of − 40 and − 26 kJ mol^−1^ at 300 K for CO_2_ and N_2_, respectively. When the calculations were performed without dispersion force correction, the Δ*H*_ads_ values were close to 0 kJ mol^−1^. This result is consistent with the fact that the interaction between the network and CO_2_ is dominated by weak dispersion forces. Furthermore, the Δ*H*_ads_ values obtained from this simulation suggested that CO_2_ was physisorbed by the network.

The optimized location of CO_2_ inside network **2** was at the center of the pore (Figure S19, Supporting Information) matching the single crystal structure (Figure 3a, Supporting Information). The CO_2_ molecule had a limited degree of mobility and was trapped in a steep potential energy well bound by the pore walls. This behavior can explain the relatively high strength of CO_2_ physisorption despite the lack of strongly interacting groups. Considering the CO_2_/N_2_ selectivity, the simulated enthalpy of adsorption for CO_2_ was 14 kJ mol^−1^ larger than for N_2_. This trend is consistent with other PCNs since the higher polarizability and quadrupole moment of CO_2_ molecule typically leads to higher interaction energies.^[^ ^20^ ^]^ However, this enthalpy difference alone was not sufficient to account for the complete lack of N_2_ uptake.

**Figure 4:**
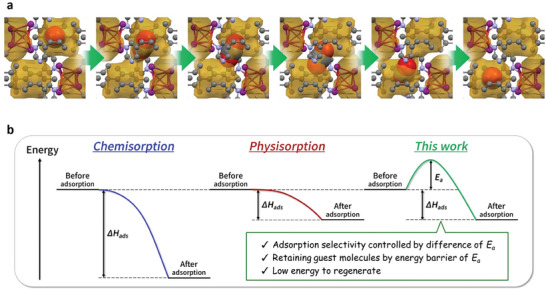
a) The diffusion path of CO_2_ through network **2** simulated by Matlantis. The accessible pore space is colored dark yellow. The traversing CO_2_ molecule is shown using space‐filling model. C – grey, N – blue, O – red, Cu – orange, and I – purple, hydrogen atoms were omitted for clarity. b) Comparison of adsorption energy diagrams for chemisorption and physisorption processes to the mechanism investigated in the present work.

Next, the diffusion path of CO_2_ through the network was evaluated. The initial state consisted of one CO_2_ molecule enclosed inside a pore of a unit cell. Whereas in the final state, the same molecule was relocated into an adjacent closed pore. The motion coordinates were linearly interpolated into seven separate intermediate states, and the Nudged Elastic Band (NEB) method^[^ ^31^ ^]^ was used to optimize their structures. The results demonstrated that CO_2_ could move from one closed pore to another without disrupting the network connectivity (**Figure** **4**a; Movie S1, Supporting Information). In the transition state, the CuI cubane cluster and the coordinated pyrimidine ring were slightly twisted, opening up a passage between the neighboring pores, which was akin to a “magic door” opening up and creating a soft stretchable channel. After CO_2_ passed through the channel, the “magic door” closed behind and the network backbone returned to the initial state. In the process, the size of the aperture increased from 1.7 × 2.1 to 2.5 × 2.6 Å in the fully open state, sufficient for CO_2_ to pass through. This transition state was more energetically unstable than the initial and final states. Therefore, for CO_2_ to diffuse through the network, it must overcome an energy barrier, which can be considered an activation energy (*E*_a_), while crossing between the pores. Similar simulations were performed for N_2_, and the *E*_a_ values for CO_2_ and N_2_ were calculated to be 55 and 67 kJ mol^−1^, respectively. The reason for this difference was attributed to the larger kinetic diameter of N_2_ (3.6 Å) compared to CO_2_ (3.3 Å).^[^ ^32^ ^]^ This effect could explain the difference in maximum uptakes of the two gases. Furthermore, since gas adsorption and desorption processes in network **2** were constrained by sizable activation energy barriers, it significantly prolonged the equilibration time between the pore and the outside environment, thus allowing the network to retain CO_2_ for extended periods.

## 3 Conclusion

In conclusion, two coordination networks with isolated voids were obtained using the same starting materials. Despite the presence of seemingly inaccessible pores in these structures, network **2** was able to selectively capture and store CO_2_. Interestingly, the structural changes before and after gas uptake were negligible, and the pores remained closed. This behavior is in stark contrast to the common gating effect reported for PCNs, where the adsorption of gases induces a dramatic phase transition, and the open and closed states can be easily distinguished and characterized. The accompanying transitions often generate significant mechanical stress that can cause irreversible damage to the network structures.^[^ ^33^ ^]^ Simulations using Matlantis revealed that the adsorption properties of **2** were controlled by what we termed a “magic door” mechanism, which facilitated channel formation between the pores through minor adjustments in the network structure allowing the passage of CO_2_ molecules. The activation energy barrier requirement for this process could rationalize the high CO_2_/N_2_ selectivity, as well as the remarkable ability of the network to retain CO_2_ while exposed to the atmosphere (Figure 4b). These results provide useful insights for future design strategies focusing on isolated voids inside PCNs that can remain sealed after gas encapsulation, thus bringing new perspectives for the development of CO_2_ sorbents.

## Conflict of Interest

The authors declare no conflict of interest.

## Supporting information

Supporting Information

Supporting Information

Supplemental Movie 1

## Data Availability

The data that support the findings of this study are available from the corresponding author upon reasonable request.
