# Supplementary material for: Long Time CO2 Storage Under Ambient Conditions in Isolated Voids of a Porous Coordination Network Facilitated by the “Magic Door” Mechanism
Source: Adv Sci (Weinh). 2023 Nov 20;11(2):2307417. doi: 10.1002/advs.202307417 (PMC10787060; doi:10.1002/advs.202307417)
Supplement: Supplementary file 1 — Supporting Information [file ADVS-11-2307417-s001.pdf]

ADVANCED SCIENCE
Open Access

Supporting Information

for *Adv. Sci.*, DOI 10.1002/advs.202307417

Long Time CO_2 Storage Under Ambient Conditions in Isolated Voids of a Porous Coordination Network Facilitated by the "Magic Door" Mechanism

Terumasa Shimada, Pavel M. Usov, Yuki Wada, Hiroyoshi Ohtsu, Taku Watanabe, Kiyohiro Adachi, Daisuke Hashizume, Takaya Matsumoto* and Masaki Kawano**

Supporting Information
©Wiley-VCH 2021
69451 Weinheim, Germany

Long time CO_2 storage under ambient conditions in isolated voids of a porous coordination network facilitated by the "magic door" mechanism

Terumasa Shimada, Pavel M. Usov,* Yuki Wada, Hiroyoshi Ohtsu, Taku Watanabe, Kiyohiro Adachi, Daisuke Hashizume, Takaya Matsumoto,* and Masaki Kawano*

Abstract: A coordination network containing isolated pores without interconnecting channels was prepared from a tetrahedral ligand and copper(I) iodide. Despite the lack of accessibility, CO_2 was selectively adsorbed into these pores at 298 K and then retained for more than one week while exposed to atmosphere. The CO_2 adsorption energy and diffusion mechanism throughout the network were simulated using Matlantis^{TM}, which helped to rationalize the experimental results. CO_2 entered the isolated voids through transient channels, termed "magic doors", which can momentarily appear within the structure. Once inside the voids, CO_2 remained locked in limiting its escape. This mechanism was facilitated by the flexibility of organic ligands and the pivot motion of cluster units. *In situ* powder X-ray diffraction revealed that the crystal structure change was negligible before and after CO_2 capture, unlike gate-opening coordination networks. The uncovered CO_2 sorption and retention ability paves the way for the design of sorbents based on isolated voids.

DOI: 10.1002/anie.2021XXXXX

Table of Contents

Table of Contents **2**
Experimental Procedures **3**
1. Instrumentation 3
Figure S1. Schematic diagram of the sample holder used for the *in situ* powder X-ray diffraction experiment. 4
2. Synthetic methods 5
Results and Discussion **6**
Figure S2. ^{1}H-NMR spectrum of 3,3′,5,5′-tetrakis(5-pyrimidyl)bimesityl (**L**) 6
Figure S3. ^{13}C-NMR spectrum of 3,3′,5,5′-tetrakis(5-pyrimidyl)bimesityl (**L**) 7
Figure S4. Single crystal structure of the as-synthesized network **1** 8
Figure S5. Single crystal structure of the as-synthesized network **2** 8
Figure S6. The UV-vis spectra 9
Figure S7. The FT-IR spectra 9
Figure S8. Powder X-ray diffraction patterns of the as-synthesized network **1** 10
Figure S9. Powder X-ray diffraction patterns of the as-synthesized network **2** 10
Figure S10. TGA and DSC data 11
Figure S11. Adsorption isotherms for network **1** 12
Figure S12. Adsorption isotherms for network **2** 13
Figure S13. Five consecutive CO_2 adsorption/desorption cycles of network **2** collected at 298 K 14
Figure S14. The FT-IR spectra of network **2** 15
Figure S15. The IR spectrum of **2@CO_2**, highlighting the 2357 – 2311 cm^{-1} region 16
Figure S16. The plot of peak area of the IR spectrum of **2@CO2** against the time 16
Figure S17. The FT-IR spectra of HKUST-1 measured in the air before and after CO_2 adsorption 17
Figure S18. Time-dependent PXRD patterns of **2@activated** exposed to CO_2 atmosphere at 100 kPa. 18
Figure S19. Optimized structure of network **2** containing CO_2 simulated by Matlantis^{TM} 19
Table S1. Crystallographic data tables (Page 1) 20
Table S2. Crystallographic data tables (Page 2) 21
References **22**
Author Contributions **22**

Experimental Procedures

1. Instrumentation

^{1}H (400 MHz) and ^{13}C NMR spectra were measured using JEOL JNM-ECA400 II spectrometer. The samples were dissolved in deuterated chloroform ($CDCl_3$) containing tetramethylsilane (TMS), and the chemical shifts were referenced against the TMS peak (δ 0.0).

Single crystal X-ray diffraction data for the ligand (**L**) and the as-synthesized networks **1** and **2** containing MeCN in the pores were measured using Rigaku VariMax X-ray diffractometer with Saturn. The crystals were cooled under nitrogen flow to 123 K using the Rigaku GNNP cryogenic cooler. Graphite-monochromated Mo Kα (λ = 0.71075 Å) was used as an X-ray source, and the detector was a two-dimensional CCD detector. The collected diffraction data were analyzed by Rigaku CrysAlisPro software.

Single crystal X-ray diffraction data for **2@activated** and **2@CO$_2$** at 90 K were measured at the BL-5A beamline, Photon Factory in the Institute of Materials Structure Science (IMSS), High Energy Accelerator Research Organization, KEK. Single crystals were cooled to 90 K by a Rigaku CryoCooler and irradiated with synchrotron radiation (λ = 0.7500 Å). Diffraction patterns were recorded by a Dectris Pilatus3 S6M detector. The measured diffraction data were integrated by XDS. For the measurements, the crystals of **2@CO$_2$** were soaked in oil immediately after taking them out of the CO_2 atmosphere.

Single crystal X-ray diffraction data for **2@CO$_2$** at 298 K was collected on a Rigaku Synergy-R/DWTI APEX II instrument with a Hypix-6000HE detector equipped with Rigaku GNNP low temperature device using PhotonJet-R X-ray source with MicroMax™-007 rotating anode Cu Kα radiation (λ = 1.54184 Å).

Adsorption/desorption isotherms of network **1** for N_2 (298 K) and CO_2 (298 K), and network **2** for N_2 (298 K) and CO_2 (273, 283, 298, 313 and 333 K) were measured using Micromeritics 3Flex Adsorption Analyzer. The powdered samples (*ca.* 70 mg) were ground in an agate mortar prior to being placed into glass sample tubes. The solvent in the pores of **1** and **2** was removed by heating the networks at 553 K and 473 K, respectively, under dynamic vacuum for 12 h using Micromeritics VacPrep 061 attached to a rotary pump. The 3Flex Version 5.03 program was used to analyze the experimental results.

The adsorption isotherms for N_2 at 77 K and CO_2 at 195 K were measured by MicrotracBEL BELSORP MAX. The adsorption volume was measured using the constant volume gas adsorption method without removing the sample cell from the apparatus. The measurement temperature was controlled by a bath of liquid nitrogen for 77 K and acetone with dry ice for 195 K. The experimental results were analyzed using the BEL MASTERTM analysis program.

Solid-state UV-vis absorption spectra were collected using a UV-Vis-NIR Spectrophotometer V-770 from JASCO Corporation (Japan Spectroscopy Co., Ltd.). The samples were ground in an agate mortar and then transferred to the sample holder. The measurements were performed in the diffuse reflectance mode using a φ = 60 mm integrating sphere unit. $BaSO_4$ white plate was used as a background. The final spectra were converted using the Kubelka-Munk (KM) transform.

Infrared absorption spectra were collected using a Nicolet™ iS™ 50 Fourier transform infrared spectrophotometer from Thermo Fisher Scientific. A liquid nitrogen-cooled MCT-A detector was used for diffuse reflectance measurements. Solid samples were diluted with potassium bromide (KBr) while pure KBr was used as the background.

TGA-DSC data was collected by Simultaneous Thermogravimetry STA449 F3 Jupiter analyzer from NETZCH. Approximately 5 mg of each sample was placed in ceramic pans and heated from room temperature to 700 °C under nitrogen flow. Before measurements, the inside of the furnace was purged with nitrogen for 10 min.

Elemental analysis (CHN) was performed using Elementar's vario MICRO cube macro-organic elemental analyzer.

Powder X-ray diffraction (PXRD) measurements were performed on a SmartLab fully automated multi-purpose X-ray diffractometer from Rigaku using Cu Kα (λ = 1.5418 Å) as an X-ray source. The powdered samples were placed between two Mylar sheets and the patterns were collected in the transmission mode on a D/teX Ultra (1D) detector.

In situ PXRD experiments under a CO_2 atmosphere were performed at the RIKEN Materials Science beamline BL44B2 in SPring-8. The measurements were carried out using a diffractometer equipped with an OHGI system[1] in transmission mode, with a wavelength of λ = 0.5201 Å. The measurement apparatus was connected to a gas cylinder and a vacuum pump via BELSORP-G, which allowed for gas introduction with controllable pressure. Powdered samples of **2@activated** were packed into glass capillaries and assembled to allow gas flow within the capillary (Figure S1). It was mounted in the apparatus, heated under vacuum at 493 K, followed by introduction of CO_2. The system was placed under approximately 100 kPa of CO_2 and the time dependent PXRD patterns were collected for 12 h at 298 K.

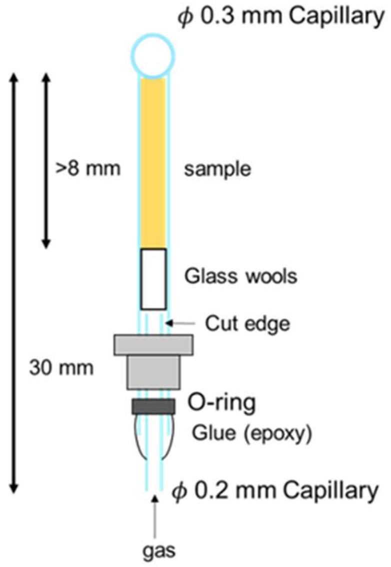

Figure S1. Schematic diagram of the sample holder used for the *in situ* powder X-ray diffraction experiment.

The calculations were carried out by PreFerred Potential with dispersion force correction (PFP+D3) version 3.0.0 on Matlantis^{TM}, a high-speed versatile atomic-scale simulator powered by a neural network potential (NNP). The initial model was a unit cell obtained from the single crystal structure and contained about 3000 atoms. By specifying periodic boundary conditions for each face of the unit cell, a complete network structure was reproduced. Using this model, the enthalpies of adsorption (ΔH_{ads}) and the diffusion path of CO_2 through the network were calculated.

2. Synthetic methods

2-1. General methods

All reagents were purchased from NARD Institute, Kanto Chemical, Tokyo Chemical Industry and Wako Chemical and used without further purification.

2-2. Synthesis of 3,3′-5,5′-tetrakis(5-pyrimidyl) bimesityl (**L**)

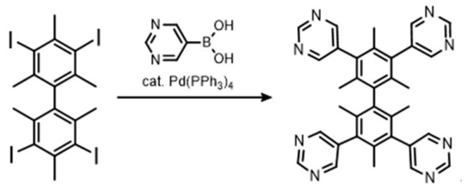

To a 100 mL Schlenk flask, tetraiodobimesityl (0.52 g, 0.70 mmol), 5-pyrimidyl boronic acid (0.41 g, 3.3 mmol), tetrakis(triphenylphosphine)palladium (0.24 g, 0.22 mmol), and potassium carbonate (2.84 g, 21 mmol) were added. Toluene (30 mL), ethanol (20 mL), and distilled water (10 mL) were degassed by bubbling N_2 for 30 min, and then transferred to the reaction flask. The reaction mixture was refluxed at 80 °C under N_2 atmosphere for 2 days. The solvents were removed by evaporation, and the residue was re-dissolved in chloroform (200 mL). The organic phase was washed with water and brine, dried over anhydrous $MgSO_4$, and evaporated to dryness to give a brown solid. The crude compound was dissolved in a small amount of chloroform and purified by silica gel column chromatography with ethyl acetate/triethylamine (100:1) as an eluent. The final product was obtained as a white powder (0.27 g, 69%). ^{1}H NMR (400 MHz, $CDCl_3$, TMS standard): δ = 9.25 (s, 4H), 8.64 (s, 8H), 1.75 (s, 6H), 1.72 (s, 12H); ^{13}C NMR (400 MHz, $CDCl_3$, TMS standard): δ = 157.8 (A), 157.3 (B), 133.6-138.7 (C-G), 20.0 (H), 18.9 (I), Elemental analysis. Calcd for [$C_{34}H_{30}N_8$·$(CH_3COOC_2H_5)_{0.25}$]: C, 73.40; H, 5.63; N, 19.56. Found: C, 73.40; H, 5.54; N, 19.34.

Single crystals of **L** for X-ray diffraction analysis were grown by slow evaporation of a chloroform solution.

2-3. Synthesis of network **1**

L (11 mg, 0.020 mmol), copper(I) iodide (19 mg, 0.10 mmol), and potassium iodide (0.83 g, 5.0 mmol) were placed inside a Teflon-lined stainless-steel autoclave. A mixture of acetonitrile (5.4 mL), distilled water (3.6 mL) and *N,N′*-dimethylformamide (DMF) (1 mL) was added and the reaction vessel was heated in an oven at 120 °C for 64 h. After that, the oven was slowly cooled to r. t. for half a day. The precipitate was removed by vacuum filtration, washed with DMF, water and acetonitrile, and dried to give a mixture of yellow plate-like (network **1**) and prism-like (network **2**) crystals. To separate the two networks, the mixed powder was immersed into a dibromomethane/dichloromethane (3:4) mixture (7 mL) inside a glass tube. After 5 min, the network **1** crystals floated to the top, whereas the network **2** crystals sunk to the bottom. The former were removed from the glass tube using a pipette and then isolated by vacuum filtration. Pure network **1** was obtained with the yield of 74 % based on **L**. The phase purity was confirmed by PXRD. Elemental analysis. Calcd for {$Cu_2I_2[C_{34}H_{30}N_8$(**L**)]·$(CH_3CN)_{0.73}$·$(H_2O)_{1.12}$}: C, 43.39; H, 3.53; N, 12.46. Found: C, 43.39; H, 3.30; N, 12.46.

2-4. Synthesis of network **2**

L (11 mg, 0.020 mmol), copper(I) iodide (30 mg, 0.16 mmol), potassium iodide (0.83 g, 5.0 mmol), and triphenylphosphine (5.2 mg, 0.020 mmol) were placed inside a Teflon-lined stainless-steel autoclave. A mixture of acetonitrile (5.4 mL), distilled water (3.6 mL) and ethanol (1 mL) was added and the reaction vessel was heated in an oven at 140 °C for 64 h. After that, the oven was slowly cooled to r. t. for half a day. The precipitate was removed by vacuum filtration, washed with DMF, water and acetonitrile, and dried to give a mixture of yellow plate-like (network **1**) and prism-like (network **2**) crystals. The same density separation method as described for the network **1** was used to purify the network **2**. After the separation was achieved, the sunken crystals of **2** were removed from glass tube using a pipette and then isolated by vacuum filtration. Pure network **2** was obtained with the yield of 58 % based on **L**. The phase purity was confirmed by PXRD. Elemental analysis. Calcd for ｛$Cu_4I_4[C_{34}H_{30}N_8$(**L**)]·$(CH_3CN)_2$·$(H_2O)_{0.71}$｝: C, 32.43; H, 2.68; N, 9.95. Found: C, 32.44; H, 2.68; N, 9.76.

2-5. Activation of network **2**

To remove the solvent guests, single crystals of network **2** (*ca.* 5 mg) were placed into a 10 mL glass ampoule, and then evacuated for 1 h. The ampoule was flame-sealed under vacuum and heated in an oven at 240 °C for 14 h. Solvent removal was confirmed by single crystal X-ray diffraction analysis, infrared spectroscopy, and elemental analysis. Elemental analysis. Calcd for {$Cu_4I_4[C_{34}H_{30}N_8$(**L**)] $(H_2O)_{1.03}$}: C, 30.68; H, 2.43; N, 8.42. Found: C, 30.68; H, 2.34; N, 8.48.

2-6. Adsorption of CO_2 into network **2**

The crystals of activated network **2** without interstitial solvent molecules were placed into a 5 mL glass vial, and a CO_2 balloon was attached through a needle. The crystals were kept in a pure CO_2 atmosphere overnight allowing a near complete saturation of the network pores. After that, the network sample was removed from the vial and further experiments were carried out under ambient atmosphere. The presence of CO_2 was confirmed by single crystal X-ray diffraction analysis and infrared spectroscopy.

Results and Discussion

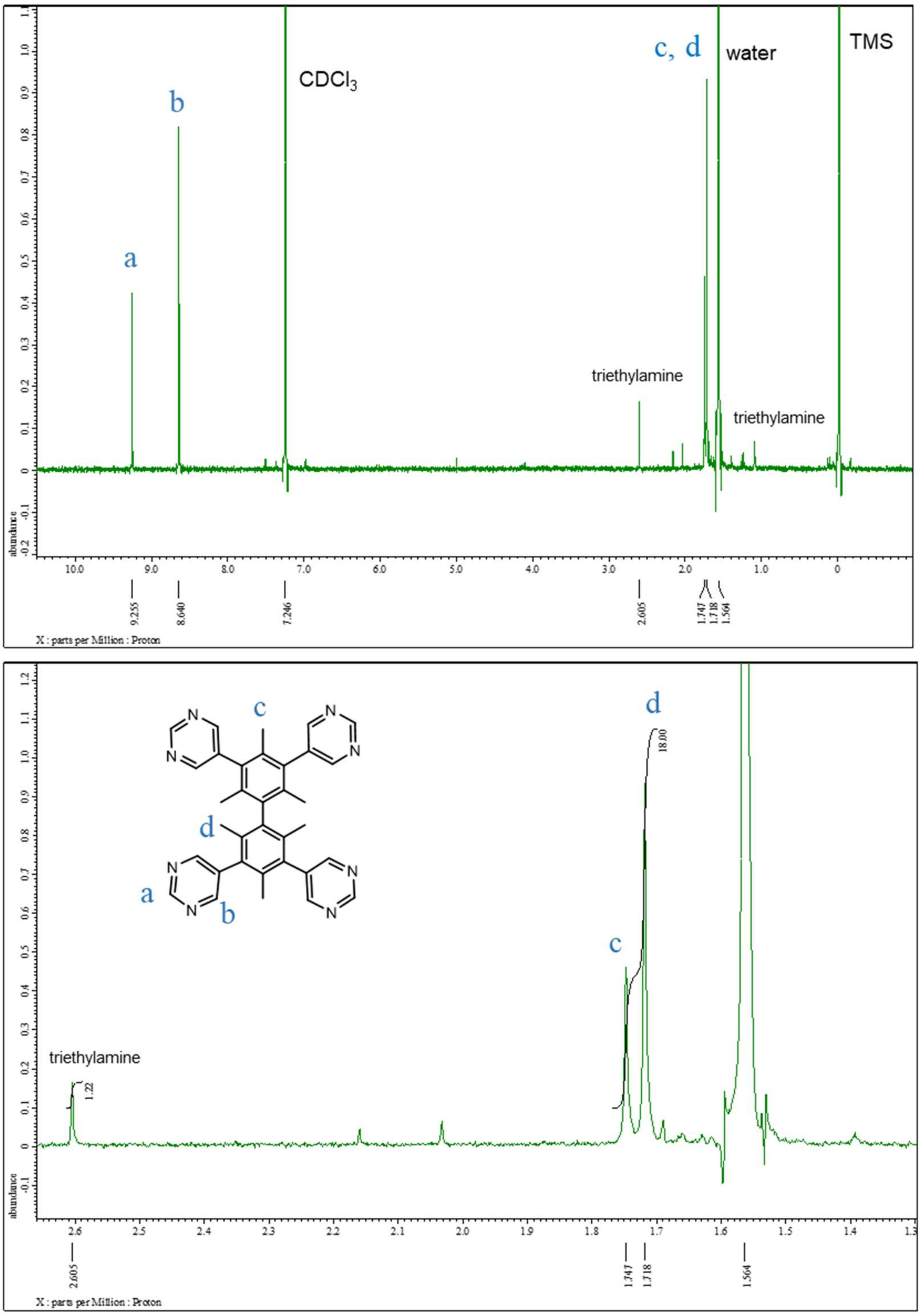

Figure S2. ^{1}H-NMR spectrum of 3,3′,5,5′-tetrakis(5-pyrimidyl)bimesityl (**L**) measured in $CDCl_3$ with TMS standard. δ9.25 (s, 4H), 8.64 (s, 8H), 1.75 (s, 6H), 1.72 (s, 12H).

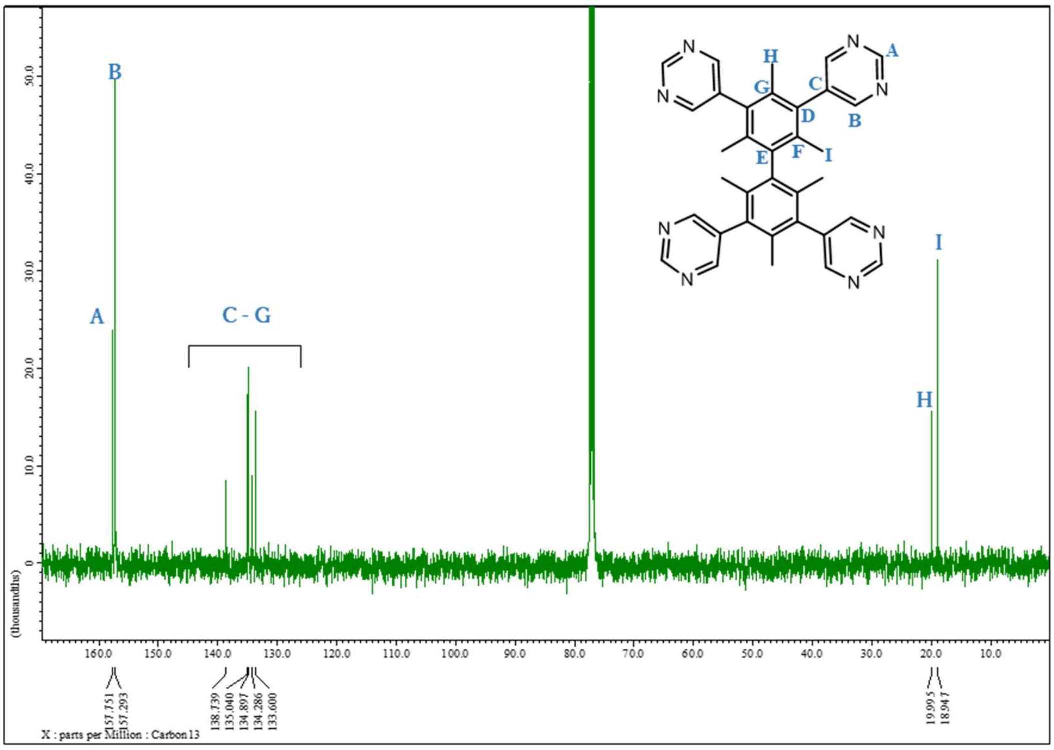

Figure S3. ^{13}C-NMR spectrum of 3,3′,5,5′-tetrakis(5-pyrimidyl)bimesityl (**L**) measured in $CDCl_3$ with TMS standard. δ157.8 (A), 157.3 (B), 133.6-138.7 (C-G), 20.0 (H), 18.9 (I).

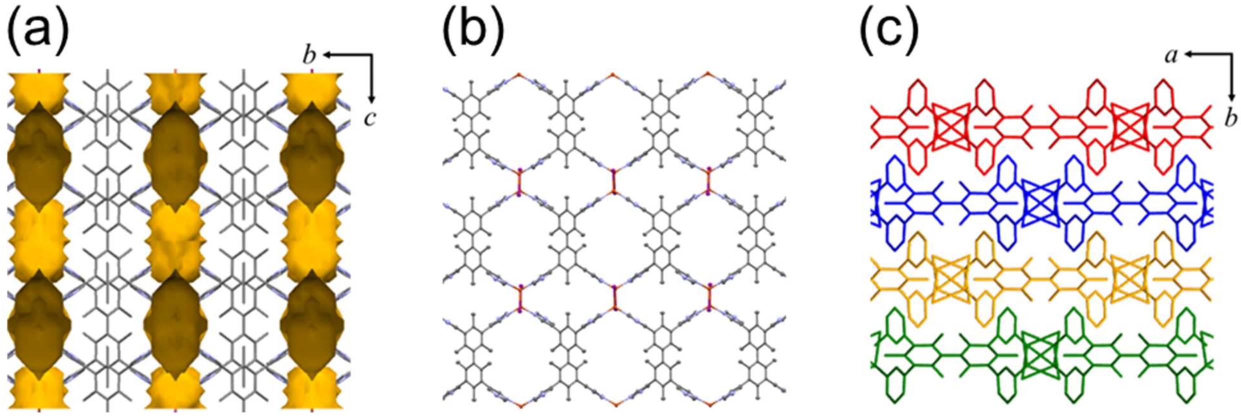

Figure S4. Single crystal structure of the as-synthesized network **1** showing a) the voids outlined in dark yellow, b) a single 2D layer and c) multiple layers represented by different colors. C – grey, N – blue, and Cl – light green; hydrogen atoms were omitted for clarity.

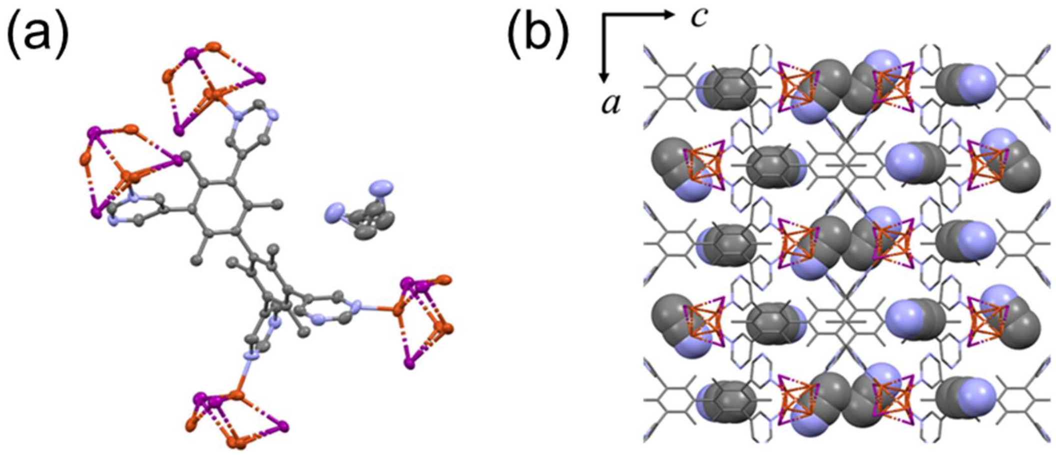

Figure S5. Single crystal structure of the as-synthesized network **2**. a) connectivity of the ligand with CuI cubane clusters and disordered acetonitrile in the pore and (b) packed structure view down the *b* axis, (acetonitrile molecules are shown using spacefilling model) C – grey, N – blue, Cu – orange, and I – purple, hydrogen atoms were omitted for clarity.

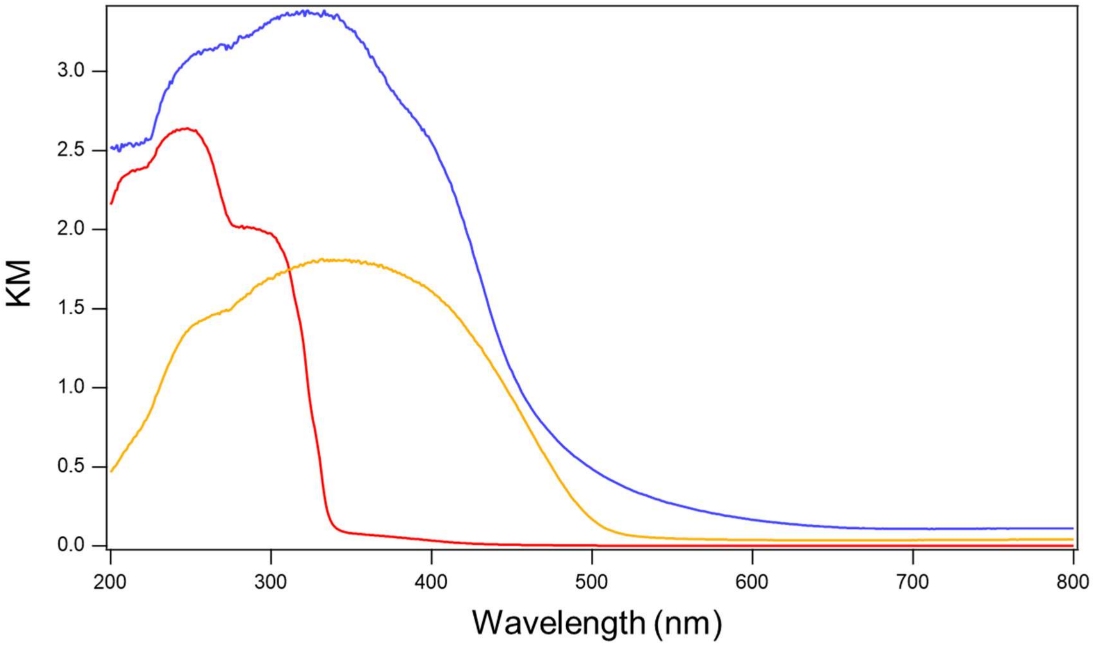

Figure S6. The solid-state diffuse reflectance UV-vis spectra (Ligand (**L**) – red, network **1** – orange, and network **2** – blue).

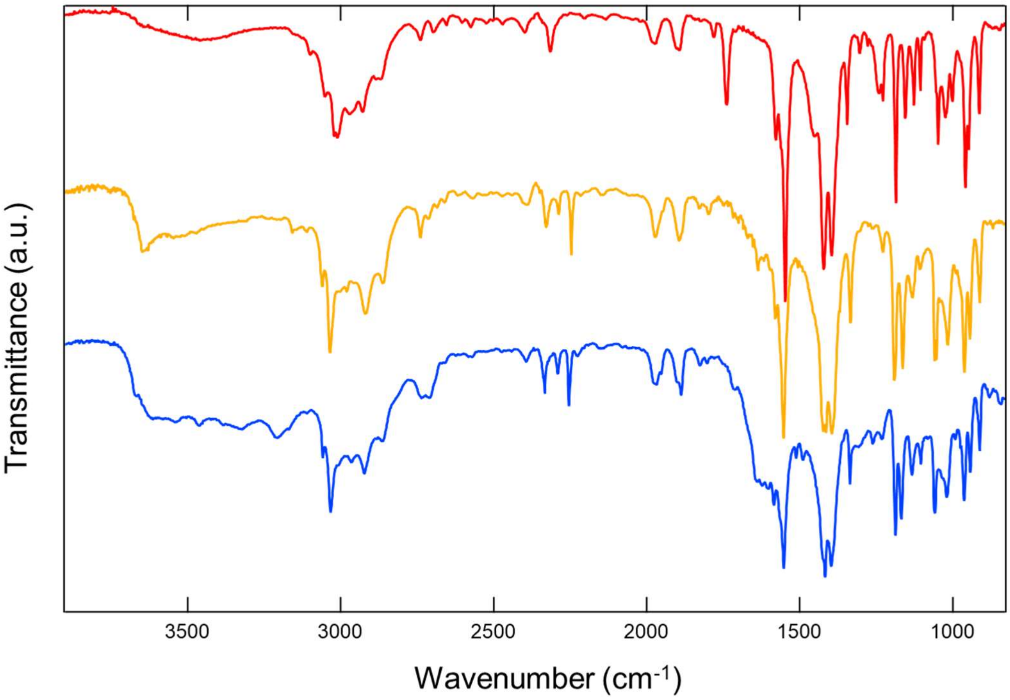

Figure S7. The solid-state diffuse reflectance FT-IR spectra (Ligand (**L**) – red, network **1** – orange, and network **2** – blue).

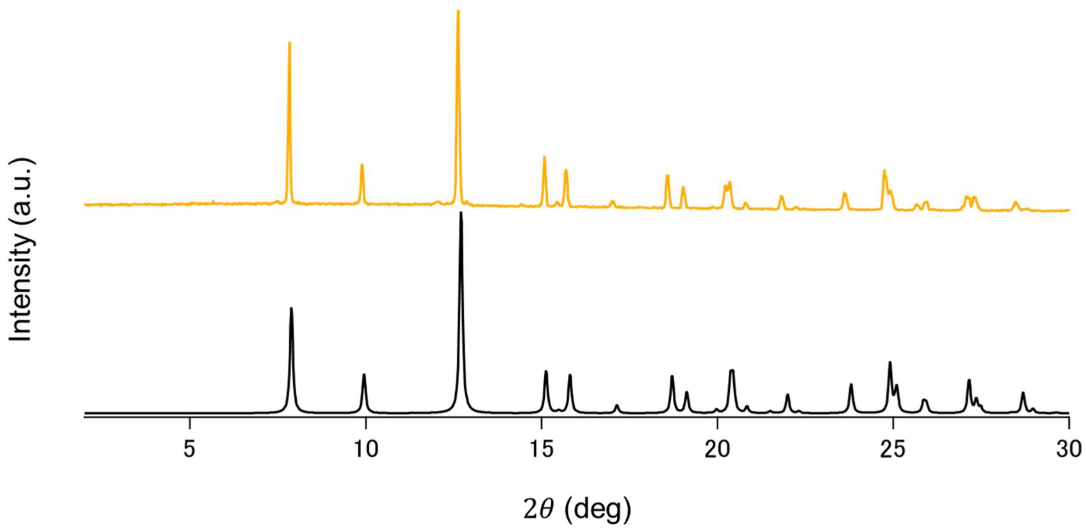

Figure S8. Powder X-ray diffraction patterns (λ = 1.5418 Å) of the as-synthesized network **1** (experimental – orange and simulated – black).

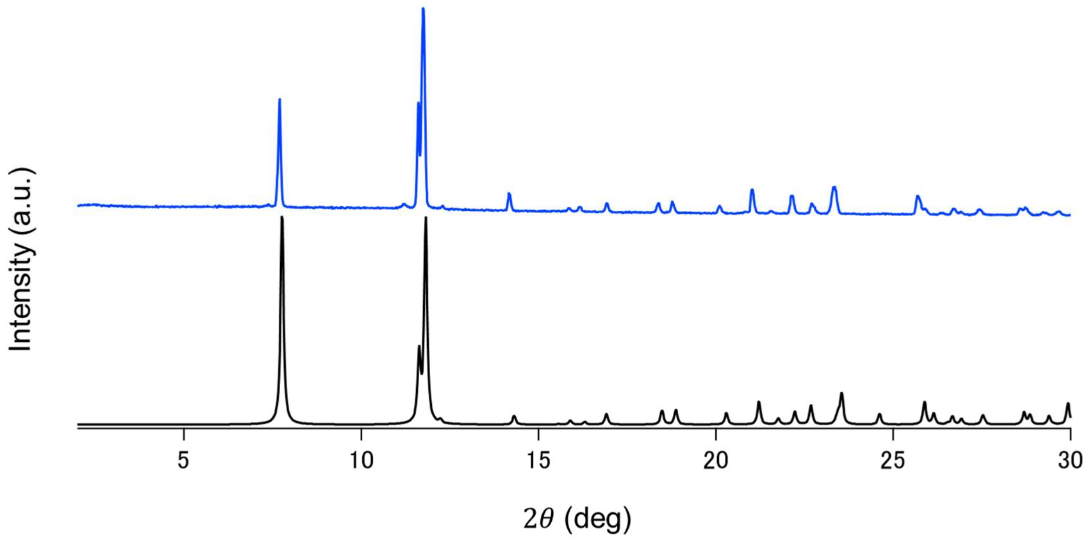

Figure S9. Powder X-ray diffraction patterns (λ = 1.5418 Å) of the as-synthesized network **2** (experimental – blue and simulated – black).

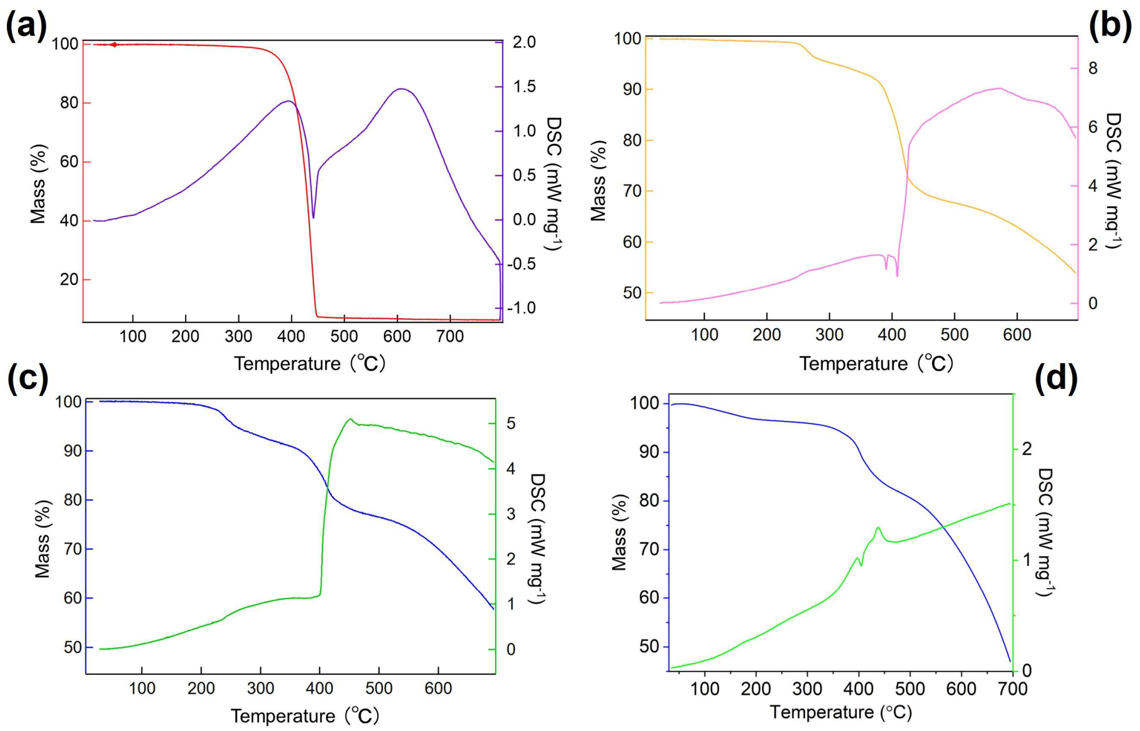

Figure S10. TGA and DSC data for a) **L**, b) network **1**, c) network **2** and d) **2@CO_2.**

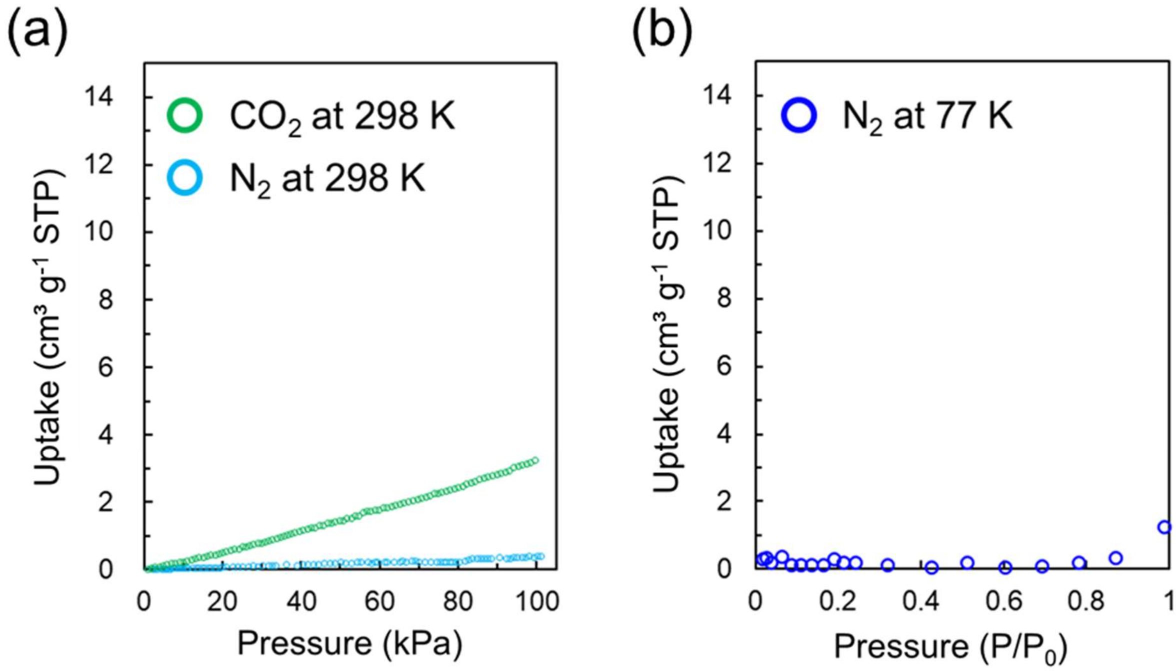

Figure S11. Adsorption isotherms for network **1** of a) N_2 (blue) and CO_2 (green) measured at 298 K, and b) N_2 (yellow) measured at 77 K.

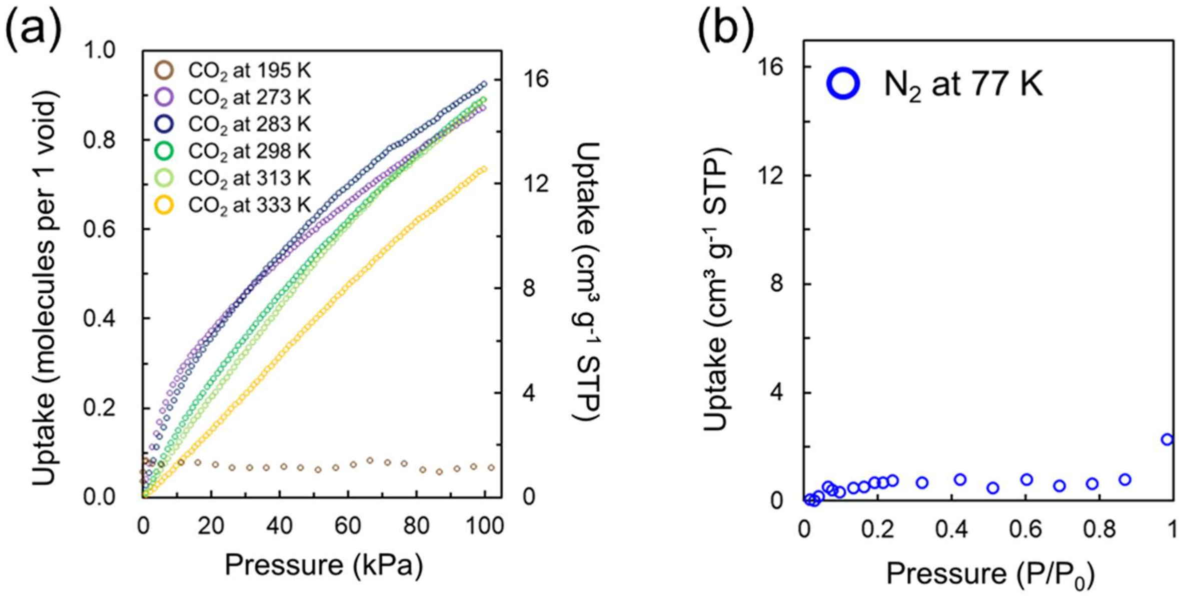

Figure S12. Adsorption isotherms for network **2** of a) CO_2 measured at different temperatures (195 K – brown, 273 K – purple, 283 K – dark blue, 298 K – green, 313 K – light green, and 333 K – yellow) and b) N_2 measured at 77 K.

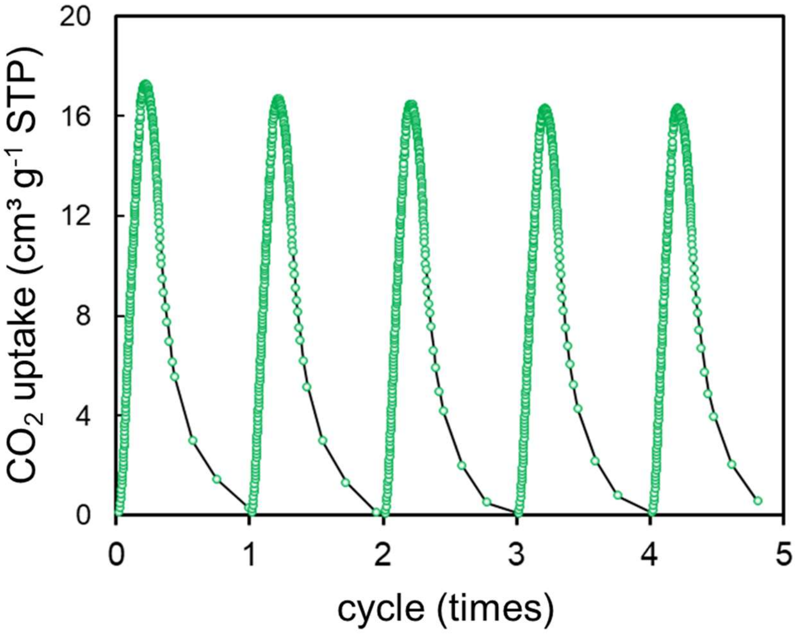

Figure S13. Five consecutive CO_2 adsorption/desorption cycles of network **2** collected at 298 K.

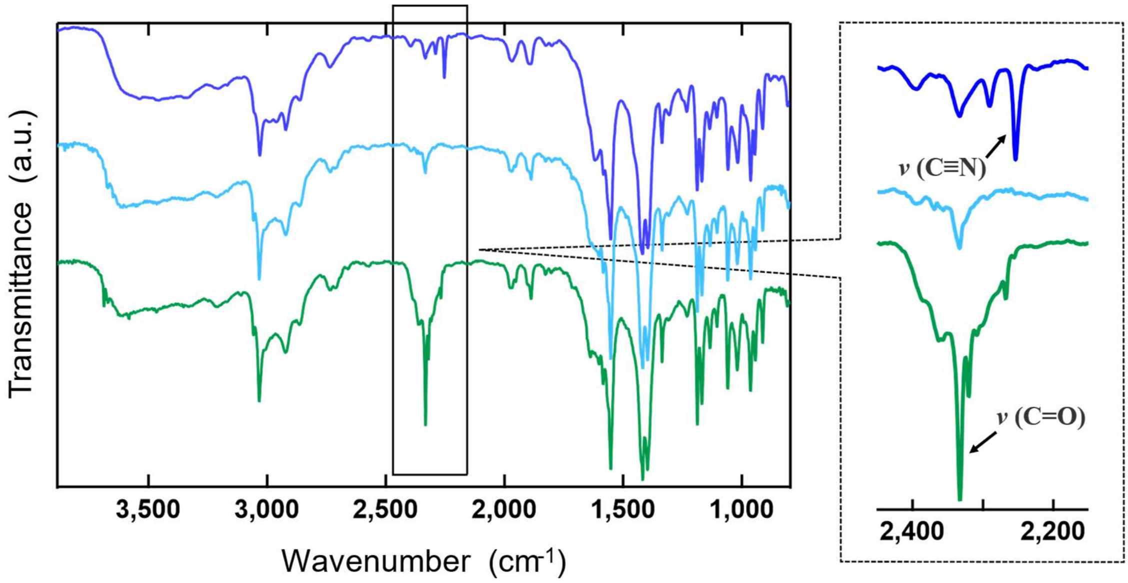

Figure S14. The solid-state diffuse reflectance FT-IR spectra of network **2** (as synthesized – blue, **2@activated** – light blue, and **2@CO$_2$** – green).

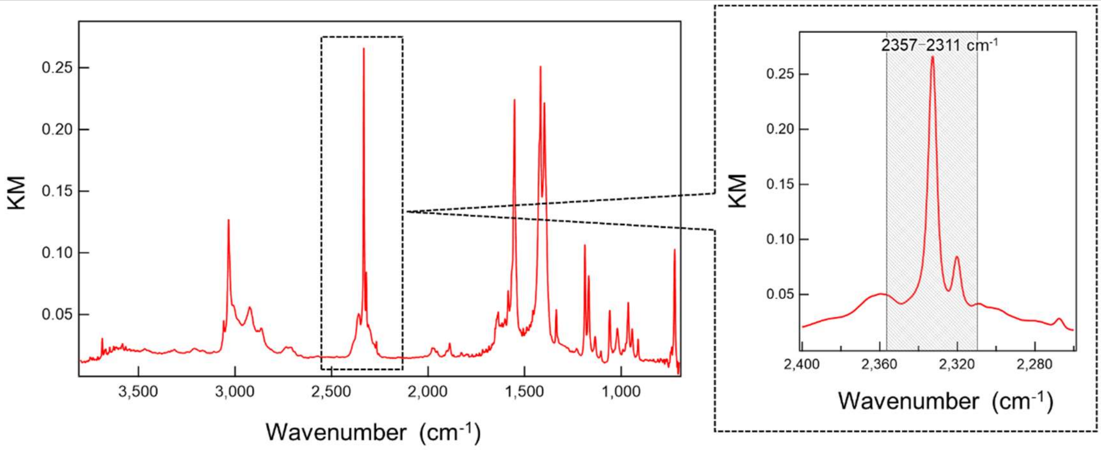

Figure S15. The IR spectrum of **2@CO₂**, highlighting the 2357 – 2311 cm^{-1} region corresponding to the adsorbed CO_2. The evolution of the peaks in this region with time was used to monitor the CO_2 release from the network.

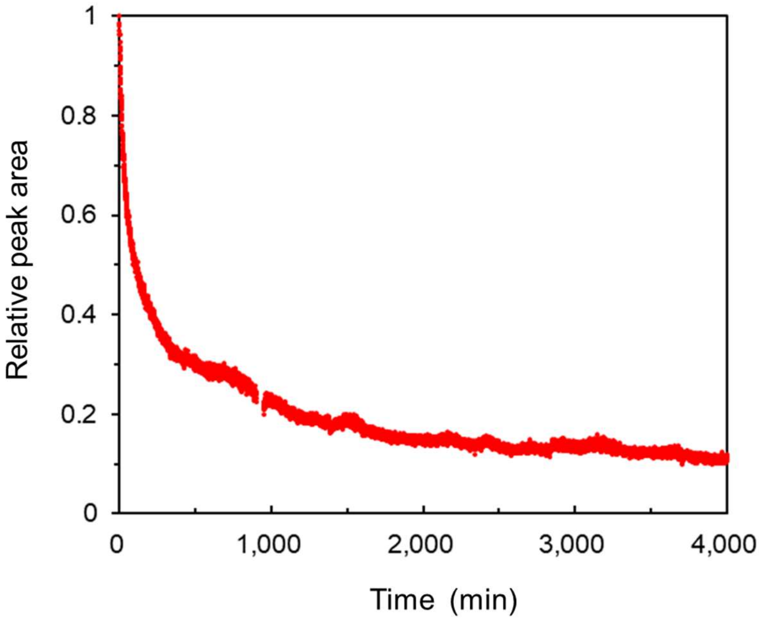

Figure S16. The plot of peak area obtained from the 2357 – 2311 cm^{-1} range of the IR spectrum of **2@CO2** against the time it was exposed to the air at 298 K.

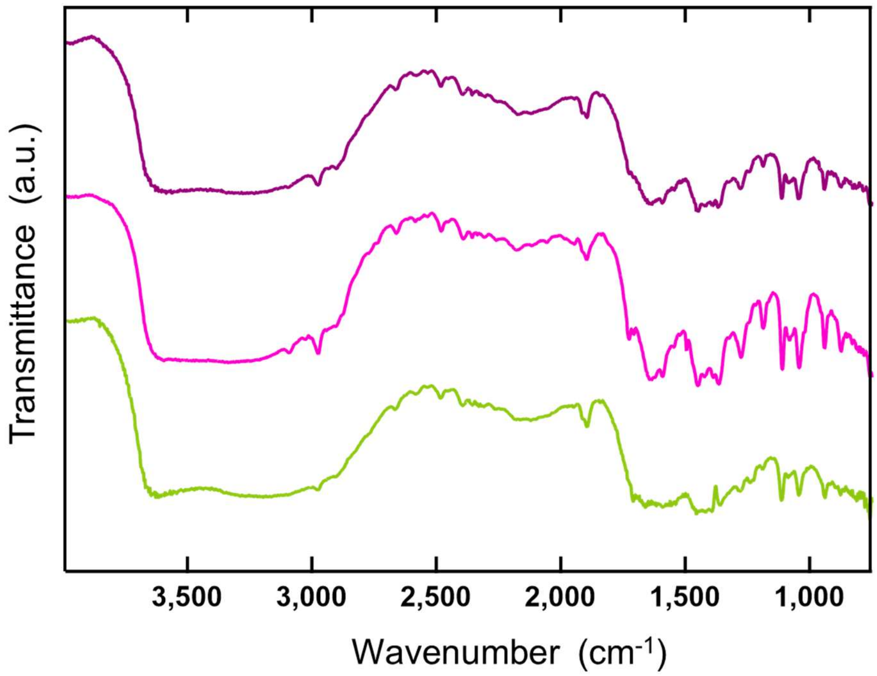

Figure S17. The solid-state diffuse reflectance FT-IR spectra of HKUST-1 measured in the air before and after CO_2 adsorption, (as synthesized – dark magenta, activated – pink, and immediately after CO_2 adsorption – light green). HKUST-1 was synthesized and activated using literature procedures.[2]

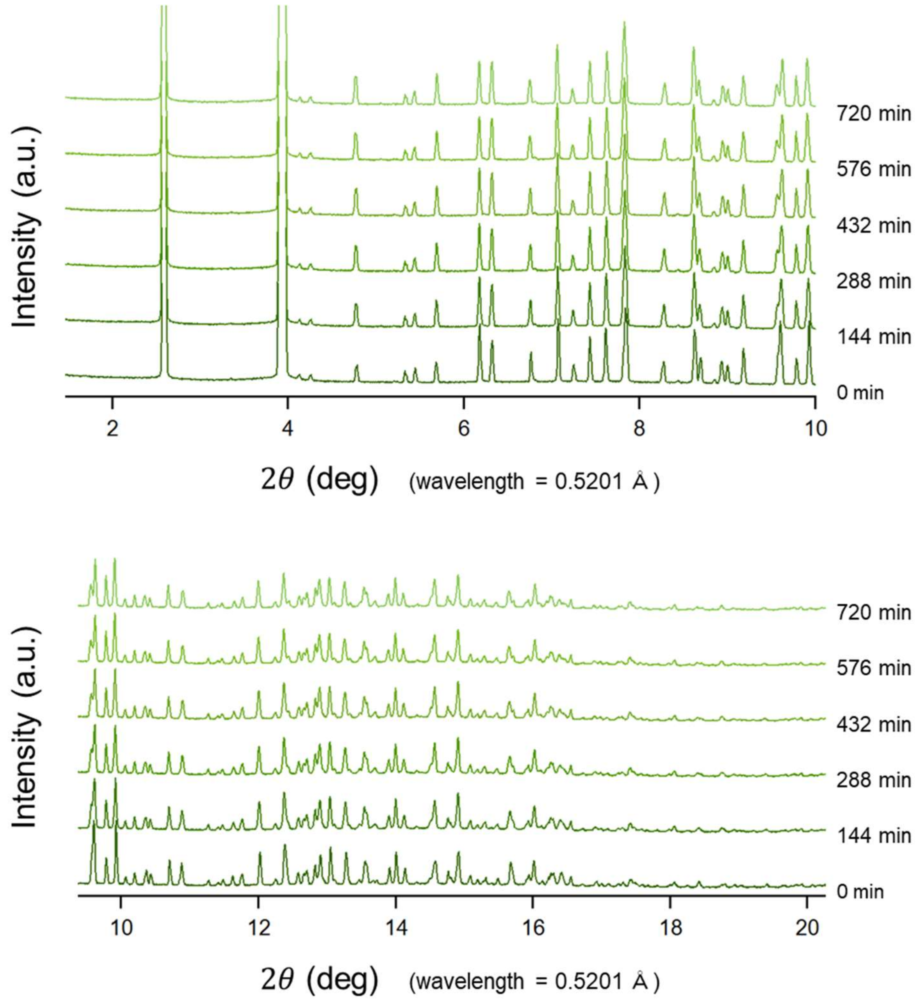

Figure S18. Time-dependent PXRD patterns of **2@activated** collected *in situ* as the network was being exposed to CO_2 atmosphere at 100 kPa.

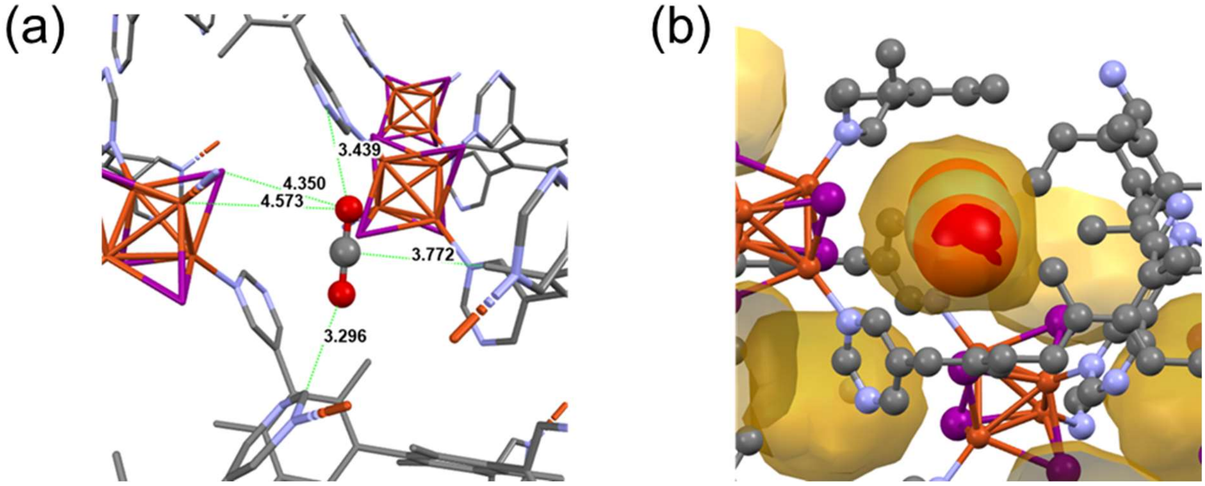

Figure S19. Optimized structure of network **2** containing CO_2 simulated by Matlantis[TM]. (a) short contacts between network **2** and the CO_2 guest, (b) CO_2 orientation inside the pore (dark yellow), C – grey, N – blue, O – red, Cu – orange, and I – purple, hydrogen atoms were omitted for clarity.

Table S1. Crystallographic data tables (Page 1).

Identification code	ligand (L)	network1	network2@synthesized
Empirical formula	$C_{36.5}H_{34.9}Cl_{7.5}N_8$	$C_{35.9}H_{30}Cu_2I_2N_{8.95}$	$C_{38}H_{30}Cu_4I_4N_{10}$
Formula weight	851.50	967.57	1388.48
Temperature/K	123	123	123
Crystal system	*orthorhombic*	*orthorhombic*	*tetragonal*
Space group	$Pna2_1$	*Ibam*	$I4_1/a$
a/Å	18.117(1)	11.6901(2)	12.3671(1)
b/Å	19.201(1)	13.7628(3)	12.3671(1)
c/Å	11.9888(8)	22.4211(5)	28.9126(7)
α/°	90	90	90
β/°	90	90	90
γ/°	90	90	90
Volume/Å^3	4170.7(5)	3607.3(1)	4422.0(1)
Z	4	4	4
ρ_{calc}/(g·cm^{-3})	1.356	1.782	2.086
μ/mm^{-1}	0.545	2.930	4.730
F(000)	1750.0	1888.0	2624.0
Crystal size/mm^3	0.107 × 0.035 × 0.014	0.285 × 0.115 × 0.049	0.257 × 0.114 × 0.094
Radiation	MoKα (λ = 0.71073)	MoKα (λ = 0.71073)	MoKα (λ = 0.71073)
2θ range for data collection/°	3.09 to 62.392	6.948 to 55.118	6.59 to 55.096
Index ranges	$-24 \le h \le 24$, $-27 \le k \le 26$, $-17 \le l \le 16$	$-15 \le h \le 15$, $-17 \le k \le 17$, $-29 \le l \le 29$	$-16 \le h \le 16$, $-16 \le k \le 16$, $-36 \le l \le 37$
Reflections collected	58408	21321	27461
Independent reflections	12584 [R_{int} = 0.2184, R_{sigma} = 0.2905]	2139 [R_{int} = 0.0285, R_{sigma} = 0.0118]	2540 [R_{int} = 0.0673, R_{sigma} = 0.0216]
Data/restraints/parameters	12584/1/520	2139/0/122	2540/0/142
Goodness-of-fit on F^2	0.983	1.099	1.224
Final *R* indexes [I>=2σ(I)]	R_1 = 0.1095, wR_2 = 0.2241	R_1 = 0.0287, wR_2 = 0.0746	R_1 = 0.0751, wR_2 = 0.1812
Final *R* indexes [all data]	R_1 = 0.3034, wR_2 = 0.3114	R_1 = 0.0304, wR_2 = 0.0754	R_1 = 0.0764, wR_2 = 0.1817
Largest diff. peak/hole / e Å^{-3}	0.95/-0.37	1.27/-0.80	1.69/-1.03
CCDC deposit number	2214828	2214829	2214830

Table S2. Crystallographic data tables (Page 2).

Identification code	network2@activated	network2@CO2_90K	network2@CO2_298K
Empirical formula	$C_{34}H_{30}Cu_4I_4N_8$	$C_{35}H_{30}Cu_4I_4N_8O_2$	$C_{34}H_{30}Cu_4I_4N_8$
Formula weight	1312.42	1356.43	1312.42
Temperature/K	90	90	298
Crystal system	*tetragonal*	*tetragonal*	*tetragonal*
Space group	$I4_1/a$	$I4_1/a$	$I4_1/a$
a/Å	12.3500(6)	12.362(1)	12.4398(1)
b/Å	12.3500(6)	12.362(1)	12.4398(1)
c/Å	28.851(2)	28.780(6)	28.8312(6)
α/°	90	90	90
β/°	90	90	90
γ/°	90	90	90
Volume/Å^3	4400.4(6)	4398(1)	4461.6(1)
Z	4	4	4
ρ_{calc}/(g·cm^{-3})	1.981	2.049	1.954
μ/mm^{-1}	5.444	5.455	24.130
F(000)	2472.0	2560.0	2472.0
Crystal size/mm^3	0.071 × 0.039 × 0.035	0.120 × 0.073 × 0.065	0.122 × 0.057 × 0.050
Radiation	synchrotron (λ = 0.750)	synchrotron (λ = 0.750)	Cu Kα (λ = 1.54184)
2θ range for data collection/°	3.786 to 71.416	3.784 to 71.352	7.74 to 152.042
Index ranges	$-15 \le h \le 15$, $-18 \le k \le 18$, $-43 \le l \le 36$	$-19 \le h \le 18$, $-19 \le k \le 19$, $-36 \le l \le 43$	$-15 \le h \le 9$, $-15 \le k \le 15$, $-32 \le l \le 35$
Reflections collected	18479	16910	8618
Independent reflections	3407 [R_{int} = 0.0436, R_{sigma} = 0.0327]	3351 [R_{int} = 0.0390, R_{sigma} = 0.0318]	2241 [R_{int} = 0.0357, R_{sigma} = 0.0287]
Data/restraints/parameters	3407/0/117	3351/0/129	2241/0/117
Goodness-of-fit on F^2	1.179	1.167	1.076
Final R indexes [I>=2σ(I)]	R_1 = 0.0468, wR_2 = 0.1481	R_1 = 0.0586, wR_2 = 0.1635	R_1 = 0.0388, wR_2 = 0.1090
Final R indexes [all data]	R_1 = 0.0551, wR_2 = 0.1742	R_1 = 0.0633, wR_2 = 0.1687	R_1 = 0.0420, wR_2 = 0.1110
Largest diff. peak/hole / e Å^{-3}	1.00/-1.69	0.78/-1.31	1.04/-0.66
CCDC deposit number	2214831	2214832	2214833

References

[1] a) K. Kato, R. Hirose, M. Takemoto, S. Ha, J. Kim, M. Higuchi, R. Matsuda, S. Kitagawa, M. Takata, *AIP Conference Proceedings* **2010**, *1234*, 875-878; b) K. Kato, H. Tanaka, *Advances in Physics: X* **2016**, *1* (1), 55-80.
[2] K. Schlichte, T. Kratzke, S. Kaskel, *Microporous and Mesoporous Materials* **2004**, *73*, 81-88.

Author Contributions

T. S.: experiments, data analysis, computational simulations, and manuscript writing, P. M. U.: conseptualization, supervision, and manuscript writing, Y. W.: single-crystal X-ray diffraction analysis, H. O.: powder X-ray diffraction analysis, and data analysis, T. W.: computational simulations, K. A.: powder X-ray diffraction analysis, D. H.: powder X-ray diffraction analysis, T. M.: conseptualization, funding acquisition, and supervision, and M. K.: conseptualization, funding acquisition, and supervision.
