# Supplementary figures and images for: Long Time CO2 Storage Under Ambient Conditions in Isolated Voids of a Porous Coordination Network Facilitated by the “Magic Door” Mechanism
Source: Adv Sci (Weinh). 2023 Nov 20;11(2):2307417. doi: 10.1002/advs.202307417 (PMC10787060; doi:10.1002/advs.202307417)

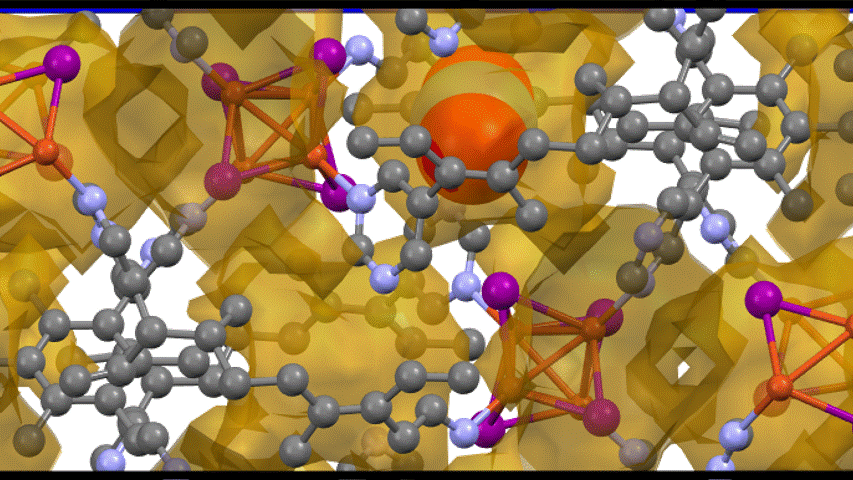

Supplement: Supplementary file 3 — Supplemental Movie 1 [file ADVS-11-2307417-s002.gif]
